# Germline Allele-Specific Expression of *DAPK1* in Chronic Lymphocytic Leukemia

**DOI:** 10.1371/journal.pone.0055261

**Published:** 2013-01-28

**Authors:** Quan-Xiang Wei, Rainer Claus, Thomas Hielscher, Daniel Mertens, Aparna Raval, Christopher C. Oakes, Stephan M. Tanner, Albert de la Chapelle, John C. Byrd, Stephan Stilgenbauer, Christoph Plass

**Affiliations:** 1 Division of Epigenomics and Cancer Risk Factors, German Cancer Research Center, Heidelberg, Germany; 2 Department of Hematology/Oncology, University of Freiburg Medical Center, Freiburg, Germany; 3 Division of Biostatistics, German Cancer Research Center, Heidelberg, Germany; 4 Cooperation Unit “Mechanisms of Leukemogenesis”, German Cancer Research Center (DKFZ), Heidelberg, Germany; 5 Department of Internal Medicine III, University of Ulm, Ulm, Germany; 6 Cancer Center, Stanford University, Stanford, California, United States of America; 7 Department of Molecular Virology, Immunology, and Medical Genetics, Human Cancer Genetics Program, Comprehensive Cancer Center, The Ohio State University, Columbus, Ohio, United States of America; 8 Department of Internal Medicine, Division of Hematology, Comprehensive Cancer Center, The Ohio State University, Columbus, Ohio, United States of America; The Ohio State University, United States of America

## Abstract

We previously reported a rare germline variant (c.1-6531) that resulted in allele–specific expression (ASE) of *death-associated protein kinase 1* (*DAPK1*) and predisposition to chronic lymphocytic leukemia (CLL). We investigated a cohort of CLL patients lacking this mutation for the presence of ASE of *DAPK1*. We developed a novel strategy that combines single-nucleotide primer extension (SNuPE) with MALDI-TOF mass spectrometry, and detected germline *DAPK1* ASE in 17 out of 120 (14.2%) CLL patients associated with a trend towards younger age at diagnosis. ASE was absent in 63 healthy controls. Germline cells of CLL patients with ASE showed increased levels of DNA methylation in the promoter region, however, neither genetic nor further epigenetic aberrations could be identified in the *DAPK1* 5′ upstream regulatory region, within distinct exons or in the 3′-UTR. We identified B-lymphoid malignancy related cell line models harboring allelic imbalance and found that allele-specific methylation in *DAPK1* is associated with ASE. Our data indicate that ASE at the *DAPK1* gene locus is a recurrent event, mediated by epigenetic mechanisms and potentially predisposing to CLL.

## Introduction

Chronic lymphocytic leukemia (CLL) is the most common leukemia of adults in the Western world with an annual incidence of 4.48 per 100.000 [1]. It is characterized by late onset with a median age of 72 years at diagnosis. The CLL genome is characterized by recurrent genetic as well as epigenetic alterations [2]. Familial clustering of CLL has been described in up to 10% of cases [3], [4]. The identification of predisposing mutations, however, has been hampered due to the lack of large pedigrees with multiple affected family members. Genome-wide association studies identified several susceptibility loci associated with CLL, however mechanisms of increased risk in carriers are largely unknown [5], [6], [7].

We have previously determined that genetic and epigenetic alterations contribute to transcriptional down-regulation of *death-associated protein kinase 1 (DAPK1)* in human CLL [8]. *DAPK1* is an actin cytoskeleton-associated calcium calmodulin-dependent serine/threonine kinase that functions as a positive mediator of both extrinsic and intrinsic apoptotic signaling pathways [9]. *DAPK1* has been demonstrated to act as a key tumor suppressor gene in CLL. Almost all cases of sporadic and familial CLL exhibit transcriptional repression associated with significantly increased DNA methylation in the *DAPK1* 5′ upstream regulatory region. Furthermore, our group reported a rare genetic variant upstream of the *DAPK1* promoter transmitted in a CLL family. This sequence variant (c.1-6531A>G) enhances the binding efficiency of the transcriptional suppressor HOXB7 to this site leading to reduced *DAPK1* mRNA expression from the affected allele resulting in allele-specific expression (ASE) [10].

In general, ASE is defined by imbalanced levels of gene expression from non-imprinted autosomal alleles [11], [12]. Several lines of evidence indicate that ASE in tumor suppressor genes may be a risk factor for the development of different cancers. Examples include ASE of the *APC* and *TGFBR1* gene which has been associated with colorectal cancer [13] or ASE of *BRCA1* and *BRCA2* in breast cancer [14]. The molecular causes of ASE are largely unknown, but may include nonsense mediated mRNA decay, variations in miRNA binding sites or other gene regulatory sequences, alternative splicing and alternative polyadenylation [14], [15], [16], [17]. Functional genomic approaches have revealed that ASE is a relatively common genome-wide phenomenon for genes and non-coding RNAs [18], [19] with estimates ranging from 5% to 10% of all genes.

Complementary to genetic alterations, accumulating evidence points to the relevance of epigenetic mechanisms for disease-associated ASE. This has convincingly been demonstrated in familial cancers where ASE is caused by heterozygous epimutation [20]. Epimutations are aberrant epigenetic marks (e.g. DNA methylation and histone modifications) inherited from one cell to a daughter cell during mitotic as well as meiotic cell division [21]. Well-characterized examples of cancer predisposing epimutations include mismatch repair genes *MLH1* [22] and *MSH2* [23] in Lynch syndrome and *BRCA1* in sporadic breast cancers [24].

In the present study, we test the hypothesis that ASE of *DAPK1* might be prevalent in cases with sporadic CLL and caused by mechanisms other than the rare sequence variant reported by Raval et al. [8]. We developed a quantitative semi high-throughput assay to measure ASE of *DAPK1* and applied this new method to test the hypothesis that ASE of *DAPK1* is both biologically and clinically significant in CLL.

## Materials and Methods

### Patient samples and sample preparation

Blood specimens from 303 patients with CLL were received from the Department Internal Medicine III, University Hospital Ulm with written informed consent and ethics approval from the Ulm University ethics committee (Ethikkommission Universität Ulm) according to the principles expressed in the Declaration of Helsinki. From 120 genetically informative patients (being heterozygous at the investigated SNPs), diagnostic samples included peripheral blood mononuclear cells (PBMC) in 110 cases and bone marrow mononuclear cells in 10 cases. CLL specimens from 36 patients including 11 genetically informative patients were obtained from the National Center of Tumor Diseases (NCT) Heidelberg for separate analysis of cell fractions negative for CLL cells (CD19 depleted). PBMC from 63 healthy donors were either derived from Ficoll density centrifugation or directly collected after 5-minute erythrocyte lysis with 1× Red Blood Cell Lysis Buffer (IMGENEX, San Diego) and used as normal controls. CD19 positive B cell fractions as well as CD19 depleted PBMC fractions (median contaminating CD19+ cells 3.5%, range 1.3–50.5%) were generated by MACS cell sorting technique following manufacturer's recommendations (Miltenyi Biotec, Bergisch Gladbach, Germany).

### Cell culture and 5-aza-2′-deoxycytidine (DAC) treatment

Granta-519 (derived from mantle cell lymphoma, MCL), MEC-1 (derived from prolymphocytic leukemia, PLL), EHEB (derived from chronic lymphocytic leukemia, CLL) and JVM-2/JVM-3 (derived from PLL) were used for *in vitro* experiments. Cell lines were obtained from the Division of Molecular Genetics of the German Cancer Research Center and are commercially available through the German collection of microorganisms and cell cultures (DSMZ). Cell line identities were confirmed for Granta-519, MEC-1 and EHEB by DSMZ. Cells were cultured in Dulbecco's MEM (Invitrogen, Darmstadt, Germany) and 10% fetal bovine serum supplemented with 4.5 g/L glucose, 2 mM L-glutamine and 1% penicillin/streptomycin, and incubated at 37°C with 5% CO_2_. 5-aza-2′-deoxycytidine (DAC) treatment was performed at the final concentrations of 1.0 µM and 1.5 µM with medium changes and re-substitution of the drug every 24 hours for 7 days.

### RNA isolation and reverse transcription

Total RNA was isolated with the TRIzol reagent (Invitrogen, Darmstadt, Germany) following the manufacturer's protocol. RNA was precipitated from aqueous phase, dissolved in DEPC-treated water and photometrically quantified. The contaminating DNA was eliminated by DNase treatment. RNA quality was assessed by the microfluidics-based Bioanalyzer platform. RNA integrity numbers (RINs) greater than seven were considered suitable for ASE analysis. First-strand cDNA was synthesized from 0.5 µg or 1 µg of DNase-treated total RNA using Superscript III reverse transcriptase (Invitrogen, Darmstadt, Germany) according to the manufacturer's instructions. Random hexamer primers (20 ng/µl final) were used for all reverse transcription (RT) reactions except for full-length *DAPK1* cDNA where oligo(dT)**_20_** primer was used (5 µM final). Non-RT reactions were included as controls. cDNA quality was verified by real-time RT-PCR for the C/EBPβ and β-actin primer set (primer sequences are given in **Supplementary Table 1**) prior to high throughput ASE detection by SNuPE/MALDI-TOF (single nucleotide primer extension/matrix assisted laser desorption ionization-time of flight) mass spectrometry.

### Genomic DNA isolation

Genomic DNA (gDNA) from cultured cells was isolated using the Puregene Core Kit A (Qiagen, Hilden, Germany) following the manufacturer's recommendations. DNA from clinical cell pellets was extracted from TRIzol lysates after RNA isolation by precipitation from interphase. DNA pellets were washed twice with 70% ethanol containing 0.1 M sodium citrate and once with 75% ethanol. Air-dried DNA pellets were re-dissolved with 8 mM sodium hydroxide and adjusted to pH 7–8 before storage.

### ASE detection by combined SNuPE/MALDI-TOF technology

Detection of ASE was based on a quantitative genotyping approach using the iPLEX Gold application (Sequenom, San Diego, USA). Multiplexed PCR was carried out to amplify four short amplicons surrounding the four exonic SNPs from cDNA and gDNA in separate reactions. Primer sequences are given in **Supplementary table 1**. PCR-based amplification was performed in 5 µl total volume in 384-well format with HotStar Taq DNA polymerase (Qiagen), a final Mg^2+^ concentration of 3.5 mM and 100 nM of each primer. Free nucleotides were inactivated by shrimp alkaline phosphatase (SAP) treatment, followed by a single nucleotide primer extension (SNuPE) reaction with four extension primers (exon 3: rs36207428-UEP, exon 16: rs3818584-UEP, exon 26: rs1056719-UEP and rs3118863-UEP) and detection of extension products by MALDI-TOF mass spectrometry. Separate distinct mass peaks represented respective alleles and peak height comparison allowed relative allele quantification. Quantitative genotyping of cDNA for ASE detection was corrected by the measured ratio of the gDNA alleles to correct/adjust for assay immanent allelic biases assuming perfectly balanced distribution. All reactions were performed in technical replicates of five or six. Multiplexed quantitative ASE measurement was validated using defined molecular standards. Plasmid based standards were generated by cloning all four pairs of *DAPK1* exonic SNPs and mixing in the following molar ratios: 50∶1, 25∶1, 10∶1, 7∶1, 5∶1, 2∶1, 1∶1, to 1∶2, 1∶5, 1∶7, 1∶10, 1∶25, and 1∶50. Finally 1×10^−4^ ng and 1×10^−6^ ng of each pair of plasmids were applied to each reaction. For gDNA-based standard, gDNAs from two donors at polymorphic position rs1056719 were mixed in the listed ratios and 30 ng of each mixture were used as input.

### Allele-specific expression (ASE) detection by Sanger sequencing

RT-PCR products covering four exonic SNPs (rs36207428, rs3818584, rs3118863 and rs1056719) of *DAPK1* were analyzed by direct or single clone sequencing to compare the expression levels between alleles. Accordingly, four primer-pairs (DEx3_F and DEx3_R for rs36207428; DEx16_F and DEx16_R for rs3818584; DEx26_Fa and DEx26_R for rs1056719; DEx26_F and DEx26_Rb for rs3118863) were used with REDTaq PCR Reaction Mix (Sigma, Saint Louis, USA) or ReddyMix (Thermo Scientific, Epsom, USA) to amplify the fragments of interest from cDNA templates. Non-RT controls where included as control for gDNA contamination. PCR products were purified by QIAquick PCR Purification (Qiagen) and directly sequenced using forward primers. To estimate the relative mRNA expression levels between 2 alleles, the purified RT-PCR products were cloned into pMOSBlue vector (GE Healthcare, Bucks, USA). Single clones were sequenced using T7 primer.

### Real-time qPCR

Real-time qPCR was performed using FastStart TaqMan mix (Roche, Mannheim, Germany) with primer pairs DAPKR2P86_F/R and NS-P8R2_F/R for the *DAPK1* 5′ and 3′ transcript region. Data was calculated from the average of the 5′ and 3′ reaction and normalized to the three house-keeping genes, *β-actin*, *GAPDH* and *HPRT* (primer sequences are given in **Supplementary Table 1**). Mono color hydrolysis probes 86, 8, 11, 60 and 73 were used, respectively.

### Bisulfite-sequencing

Bisulfite treatment of gDNA was performed using the EZ DNA Methylation Kit (Zymo Research Corporation, Irvine, U.S.A.) according to the manufacturer's instructions. Bisulfite-treated DNA (BT-DNA) was stored at −70°C and repetitive thawing was avoided. PCR amplification was carried out using 1 µl BT-DNA template in 10–20 µl total volume. Primer sequences are given in **Supplementary Table 1**. PCR products were purified with QIAquick Gel Extraction Kit (Qiagen) and consecutively cloned using TOPO TA cloning (Invitrogen). Single clones were sequenced and evaluated using BISMA [25] and BIQ Analyzer [26] software packages.

### Quantitative DNA methylation assessment by MassCleave technology

Quantitative DNA methylation analysis at single CpG units was performed using the MassCleave application as previously described [27]. Briefly, bisulfite-treated genomic DNA was PCR-amplified, *in vitro* transcribed, cleaved by RNaseA and subjected to MALDI-TOF mass spectrometry. Primer sequences for PCR amplicons are listed in **Supplementary table 1**. Methylation standards (0%, 20%, 40%, 60%, 80% and 100% methylated whole genome amplified genomic DNA) and correction algorithms based on the R statistical computing environment were used for data normalization.

### Detection of allele-specific methylation (ASM) by SNuPE

Detection of ASM was performed using SNuPE as described above. Here, bisulfite-converted DNA was used as PCR template and methylated/unmethylated sequences were amplified separately using specific primers. Extension primer for rs13300553 was used to determine the genotype distribution between specifically amplified methylated and unmethylated alleles and one CpG dinucleotide methylation control site was interrogated for methylation status specific amplification (primer sequences are given in **Supplementary table 1**).

### Statistics

Cases and controls were tested for difference in location and variability of allele frequencies with the Mann-Whitney test and the F-test. According to recent work [13], we applied the Youden index to determine allele frequency cut-offs between cases and controls in order to identify cases with ASE. Since ASE occurred bi-directional this was done in either direction. Complementary, we used the α-outlier region approach [28] to identify ASE-positive cases outside normal allelic variation as defined by the control cohort assuming an underlying normal distribution. Observations that differed strongly in location (accounting for the scale of the distribution) were labeled as outliers using α critical value at level a. Huber's M estimator was used to obtain robust estimators for location and scale which are unaffected by small to moderate amounts of outliers. The outlier region was computed at α-level 5%. P-values below 0.05 were considered statistical significant. All calculations were carried out with R 2.13 [29].

## Results

### Development and establishment of a semi high-throughput method for ASE detection

**Figure 1:**
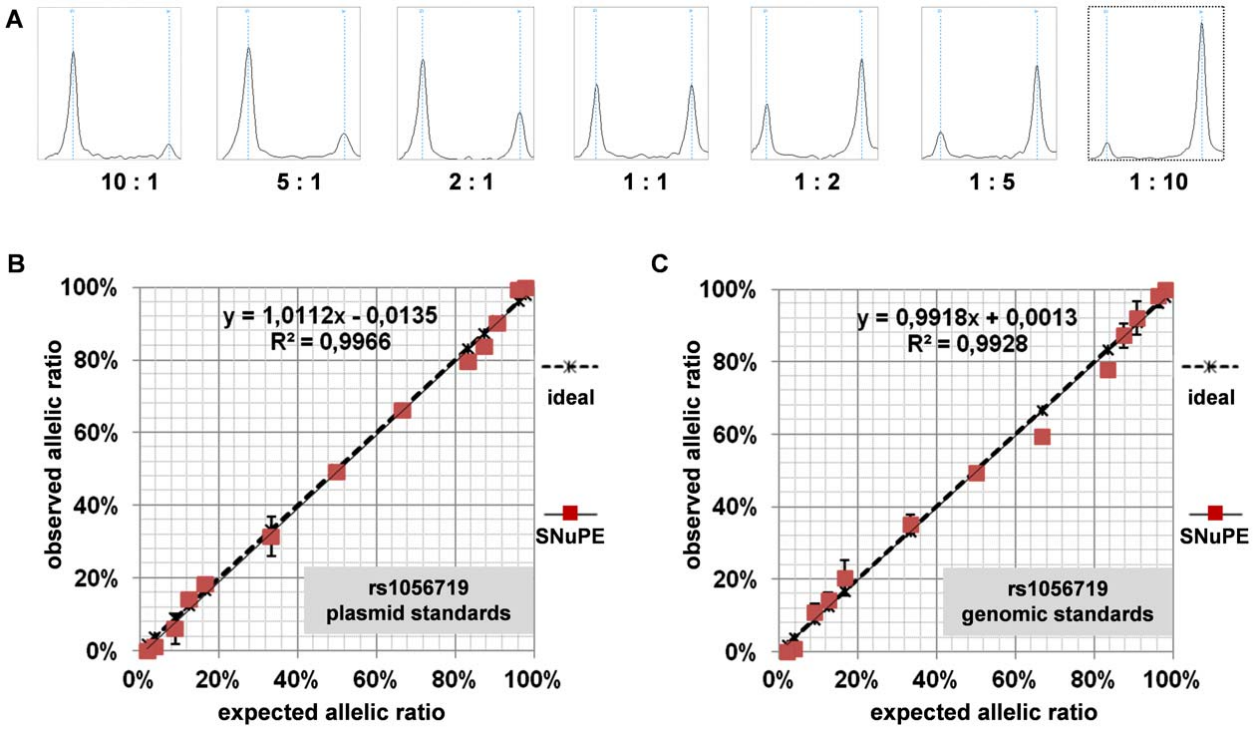
Characterization of a multiplexed MassARRAY-based method for detection of allele-specific expression (ASE). (A) Representative MassARRAY spectra of molecular standards of *DAPK1* exonic SNP rs1056719 (G/A). Each spectrum represents the mass range from 5390 to 5500 Da displaying SNP rs1056719 out of a multiplexed assay. Left peaks represent the G allele, right peaks represent the A allele. Copy number ratios between standard plasmids containing G and A alleles are given below each spectrum. (B) Standard curves for a plasmid-based standard displaying allelic ratios from 1∶50 to 50∶1 and correlation with idealized ratio (SNP rs1056719). The correlation was calculated using the Pearson correlation coefficient. (C) Standard curves for a genomic DNA based standard displaying allelic ratios from 1∶50 to 50∶1 and correlation with the idealized ratio (SNP rs1056719).

Previously, we demonstrated that both increased promoter methylation and a rare germline variant (c.1-6531A>G) at the *DAPK1* gene locus are associated with *DAPK1* transcription and thereby contribute to CLL risk. This single nucleotide variation resulted in allele-specific expression of *DAPK1* in germline, non-tumor tissue (skin-derived fibroblasts). Here we hypothesized that ASE of *DAPK1* could be present in CLL patients in the absence of this particular rare genetic variant. To test this hypothesis, a sensitive and quantitative methodology for measurement of ASE high throughput capability was developed. We combined SNuPE and MALDI-TOF mass spectrometry utilizing the iPLEX assay by Sequenom for quantitative genotyping of cDNAs (**Figure 1A**). Accuracy and reproducibility with R^2^>0.99 could be demonstrated on plasmid standards with defined SNP ratios as shown for the exonic SNP rs1056719 exhibiting highest heterozygosity frequencies (**Figure 1B**) as well as other informative *DAPK1* exonic SNPs (**Figure S1**). In order to test assay sensitivity, plasmid dilutions ranging from 30,000 down to 300 copies were analyzed (**Figure S2**). Standard deviations of four repeated measurements were below 2% in the high template sample (300000 template copies) and below 6.6% in the low template amount sample (300 template copies) indicating high detection sensitivity and robust detection even with minute template amounts. To test whether the assay accuracy was also stable for genomic DNA, defined mixtures of genomic DNA were used (**Figure 1C**). Consistent high accuracy and robustness indicated that template complexity did not affect the assay performance. Thus, the combination of SNuPE and MALDI-TOF mass spectrometry proved to be a suitable sensitive and precise tool for the quantification of *DAPK1* ASE in large cohorts.

### DAPK1 ASE occurs in CLL cases and is associated with increased promoter methylation

**Figure 2:**
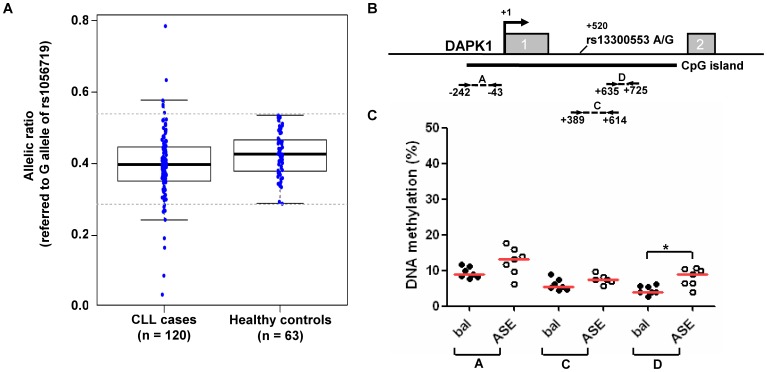
*DAPK1* allele-specific expression (ASE) in CLL patients. (A) 120 CLL cases and 63 controls were analyzed for *DAPK1* ASE using the informative SNP rs1056719 (G/A) as outlined previously. Allelic ratios (in relation to the G allele) of *DAPK1* mRNA from peripheral blood mononuclear cells (PBMCs) were measured with the outlined SNuPE/MALDI-TOF-based method. Dashed lines mark statistically (Youden index outlier method) determined thresholds to identify ASE positive outliers contributing to the significant variability of CLL compared to healthy controls. The centre of these thresholds (0.29 and 0.54, dashed lines) is the estimated average (0.4) of the CLL sample group. (B) Scheme of the *DAPK1* promoter region with grey boxes representing the first 2 exons of *DAPK1*. Nucleotide positions are given relative to *DAPK1* transcriptional start site (TSS). Dashed lines represent positions of the investigated regions/amplicons. (C) Quantitative DNA methylation analysis in the amplicons A, C and D (as described in figure 4) for the seven most imbalanced and seven most balanced CLL patients with regard to *DAPK1* mRNA expression. Scatter plots represent mean amplicon methylation levels. Significance was assessed by non-parametric Mann-Whitney-U test (* indicates p<0.01).

Next we addressed whether *DAPK1* ASE is a common feature in CLL patient samples. Out of a total of 303 patient samples that were screened from the biobanks of the University Hospital Ulm, 120 (39.6%) were identified to be informative (heterozygous) for SNP rs1056719. Out of 144 healthy donors, 63 (43.7%) displayed heterozygosity. This polymorphism showed the highest rate of heterozygosity among the four investigated *DAPK1* exonic SNPs. All 120 informative patient samples were found to be negative for the previously detected rare germline mutation at the HOXB7 binding site c.1-6531, upstream of the transcriptional start site (TSS). We analyzed whole PBMCs from 120 CLL patients and 63 healthy controls for ASE of *DAPK1*. As *DAPK1* has been shown to be consistently silenced in B cells of the CLL clone [8] and to be strongly expressed from monocytes and natural killer (NK) cells, the observed allelic expression differences can be attributed to germline in both, healthy controls and CLL patients. Variability of distributions between CLL cases and controls as assessed by F test was different as triggered by outliers among the group of CLL patients (p = 0.0002) (**Figure 2A**). We calculated lower and upper cut-offs to identify case outliers based on the Youden index (lower limit = 0.29, upper limit = 0.54). Consequently, 17 out of 120 CLL samples (14.2%) were identified to harbor allele-specific mRNA expression imbalance for *DAPK1*. Complementing this finding, we used an alternative procedure based on the α-outlier region approach at an α-level of 5% [30] to define ASE-positive patient samples stringently assuming an underlying normal distribution. Huber's M estimator was used to get robust estimators for location and scale which are unaffected by small to moderate amounts of outliers (location estimator = 0.40; scale estimator = 0.08; limits of alpha outlier region, lower = 0.25, upper = 0.55). Here, 10 out of 120 CLL samples (8.3%) with allele-specific mRNA expression imbalance for *DAPK1* were identified. The imbalance, as assessed by both approaches, resulted from the shift to either allele and is therefore bi-directional. Notably, allele frequencies were significantly different between CLL cases and healthy controls (median G vs. A ratio of 0.4 vs. 0.43 respectively, p = 0.02). To control for confounders of allelic expression balance potentially introduced by contaminating CLL cells, we tested the non-B cell fraction of the enriched mononuclear cells (referred to as “flow-through”). CLL negative fractions of PBMC were generated by CD19 depletion in 11 informative CLL patients. FACS, detecting contaminating CLL cell populations less than 2%, assured the efficacy of CD19 depletion in these CLL patient samples. A similarly pronounced widespread distribution of allelic imbalances could be observed (**Figure S3**), indicating that *DAPK1* ASE occurs in non-malignant cell populations and thus it is likely to be a germline feature.

By comparing the baseline clinical characteristics of the 14 imbalanced to the 30 most balanced CLL cases, we could not observe any statistically significant differences between the two groups. However, the age at diagnosis showed a clear trend towards earlier onset in the ASE positive group (median age = 53.0, range = 40–61 years) compared to the balanced cases (median age = 62.5, range = 41–76 years, p = 0.044). No differences in survival endpoints (overall survival, time to treatment failure) or other relevant disease characteristics known to predict prognosis (IGHV mutational status, cytogenetics) were detected.

### An epigenetic cause of DAPK1 ASE in CLL

ASE could potentially be explained by different mechanisms. Sequence analysis of all *DAPK1* exons in 96 CLL patient samples showed the presence of previously reported SNPs in exons 3, 4, 16 and 26. However, no mutations in the coding sequence that may result in nonsense mediated RNA decay were revealed. No modifications were identified in the 3′ ‘UTR of *DAPK1* that might interfere with (or create new) miRNA binding sites. We also investigated a −7 kb to +2 kb region around the *DAPK1* transcriptional start site for a haplotype associated with allelic expression imbalances. This region constitutes a genomic block with high genetic linkage disequilibrium. Fifteen SNPs were genotyped in ASE positive and negative CLL samples, however, no segregation of a genotype/haplotype with the presence of ASE could be detected. To search for evidence that epigenetic alterations (e.g. promoter methylation) are responsible for differences in germline allelic expression we first determined if promoter methylation levels are altered between germline samples from patients that showed a balanced expression and those that showed ASE. We selected seven samples from each group and measured the DNA methylation levels (**Figure 2B, C**). Interestingly we determined a trend of increased methylation in samples from CLL patients with ASE, suggesting that epigenetic mechanisms might contribute to this phenomenon. Analysis on the single CpG level identified several CpG units with significant differences in the intron 1 region (amplicon D, p<0.01). However, this analysis did not allow us to investigate DNA methylation on individual alleles.

### CLL relevant cell lines exhibit DAPK1 ASE

**Figure 3:**
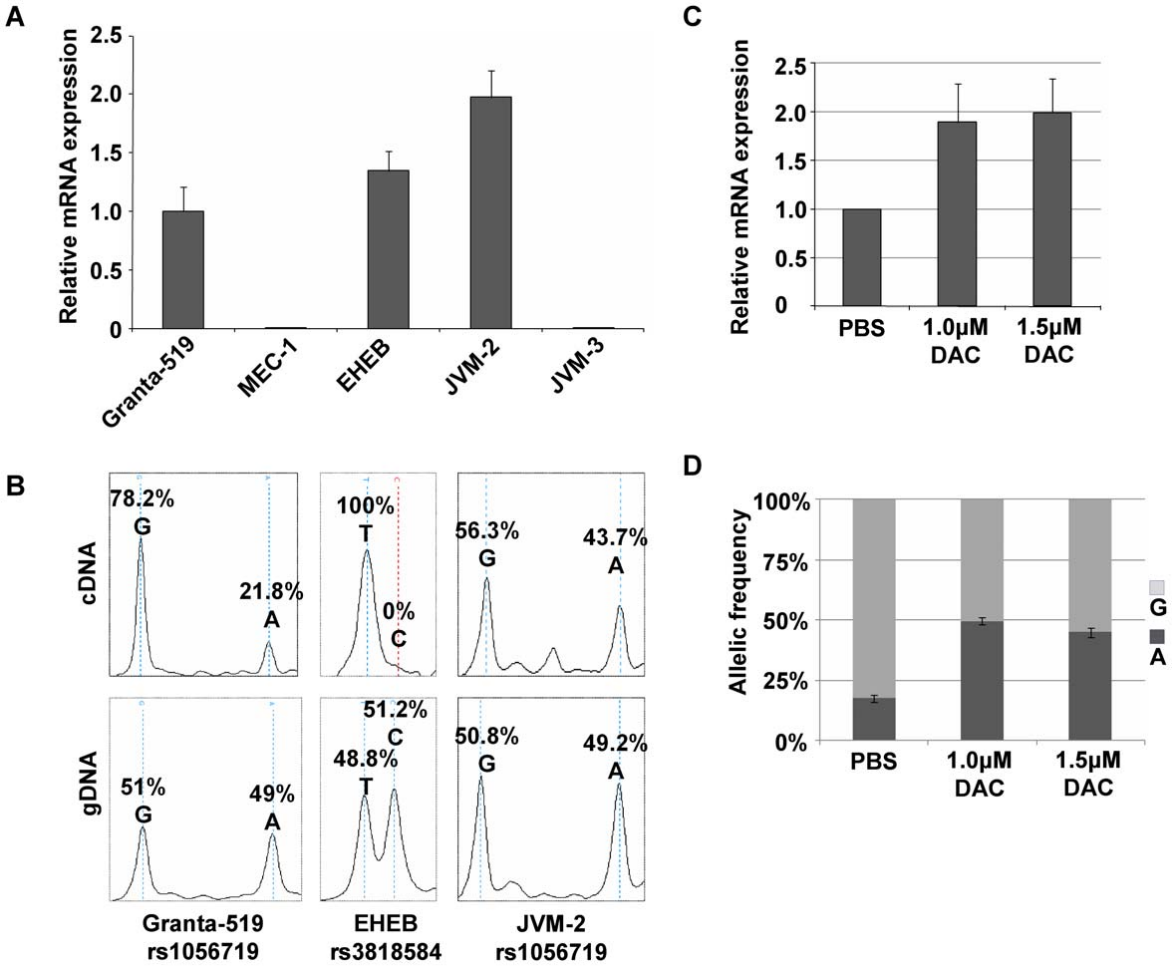
Allele-specific expression (ASE) of *DAPK1* is prevalent in B-cell malignancy derived cell lines. (A) TaqMan real-time PCR of cDNA from five B-cell malignancy cell lines (Granta-519, MCL; MEC-1, B-PLL; EHEB, chronic B-cell leukemia, JVM-2, B-PLL; JVM-3, B-PLL) show relative expression levels of *DAPK1* mRNA expression normalized to three house-keeping genes. (B) Allelic ratios of cDNA and gDNA are quantified by SNuPE/MALDI-TOF. Representative spectra demonstrate an imbalanced allelic mRNA expression compared to allelically balanced gDNA. Molecular weight range 5390–5500 Da is displayed for rs1056719 (G/A) in Granta-519 and JVM-2 cells and 6985–7045 Da for rs3818584 (T/C) in EHEB. Peak height/signal intensity correlates with allele abundance. (C) qPCR-based *DAPK1* mRNA quantification upon treatment with the DNMT inhibitor (5-aza-2′-deoxycytidine, DAC) in Granta-519. The values are relative to the respective mock control (PBS). (D) Allelic ratios of SNP rs1056719 in Granta-519 cells measured by SNuPE/MALDI-TOF assay upon DAC treatment.

To functionally assess the impact of differential methylation on ASE at the *DAPK1* gene locus, we used five human B cell lines (MEC-1, Granta-519, EHEB, JVM-2 and JVM-3) for *DAPK1* expression and promoter methylation analysis (**Figure 3A**). The overall *DAPK1* expression levels varied strikingly among these cell lines. JVM-3 and MEC-1 cells did not show detectable *DAPK1* mRNA levels. This was in concordance with markedly increased DNA methylation at the *DAPK1* promoter region in MEC-1 (**Figure S4**) reflecting the epigenetic silencing of *DAPK1* in B cells as previously shown [8]. The other cell lines showed variably low levels of *DAPK1* mRNA expression (compared to primary monocytes) and were therefore candidates for ASE. Four common exonic SNPs (rs36207428, rs3818584, rs3118863 and rs1056719) were analyzed in multiplexed reactions. Granta-519 cells showed imbalanced DAPK1 expression between the two alleles (Figure 3B). Allele-specific mRNA (cDNA) levels were considerably lower for the A allele compared to the G allele (21.8% vs. 78.2%). A balanced allelic ratio at the germline DNA level as demonstrated by equal sized spectrum peaks for A and G (49% vs. 51%) at SNP rs1056719 excluded imbalanced copy number variation at this site. The dominance of the G allele over the A allele in Granta-519 was confirmed by two additional experiments. First, single-clone sequencing of ligated PCR products generated only two out of 12 (17%) clones carrying the A allele while 10 clones were derived from the G-allele (**Fig. S5A**). Furthermore, direct sequencing electropherograms of Granta-519 cDNA and gDNA illustrated a dominance of the G- over the A allele in cDNA while both electropherogram peaks were of similar height in the gDNA (**Fig. S5B**). Similar to Granta-519, the EHEB cell line was heterozygous at exonic SNP site rs3818584 (T = 48.8% vs. C = 51.2%). However, cDNA genotyping displayed the presence of only the T allele (100% vs. 0%) indicating monoallelic mRNA expression (**Figure 3B**). Sequencing chromatograms also confirmed these results (**Figure S5C**).

### DAPK1 ASE is associated with allele-specific promoter methylation (ASM) in Granta-519 cells

To determine the cause of ASM of *DAPK1* in Granta-519, we performed sequence analysis of the genomic region extending from 5.5 kb upstream of the TSS to the end of exon 2 of the *DAPK1* gene. We identified heterozygosity for eight known SNPs (rs11141848, rs10746814, rs1035262, rs13296984, rs1035261, rs1035260, rs1964911, and rs13300553) indicating that these cells carried no deletions around the promoter region. No further genetic variation between the two alleles could be detected. Thus, we speculated that epigenetic aberrations might contribute to *DAPK1* ASE in Granta-519. To functionally test the impact of promoter methylation patterns on *DAPK1* transcription balance, we treated Granta-519 cells with different concentrations (1.0 and 1.5 µM) of the DNA methyltransferase inhibitor 5-aza-2′-deoxycytidine (DAC) for 7 days. We hypothesized that inhibition of DNA methylation might lead to potential reactivation of the repressed allele. A twofold upregulation of DAPK1 transcription was observed by qPCR (**Figure 3C**). This finding is in concordance with a reactivation of the silent allele contributing to overall expression. Simultaneously this upregulation was accompanied by a reconstitution of the allelic balance, as illustrated by the equal size of the G and A peak heights at the SNP site rs1056719 in cDNA and shown by mass spectrometry and conventional cDNA sequencing (**Figure 3D**, **Figure S6A, B**). The mock-treated control (PBS) retained imbalanced mRNA expression, and expression did not increase. Importantly, after withdrawal of DAC and one month of continued culturing, *DAPK1* ASE reappeared with an identical reduction in cDNA of the A allele relative to the G allele compared to untreated Granta-519 cells (**Figure S6C**).

**Figure 4:**
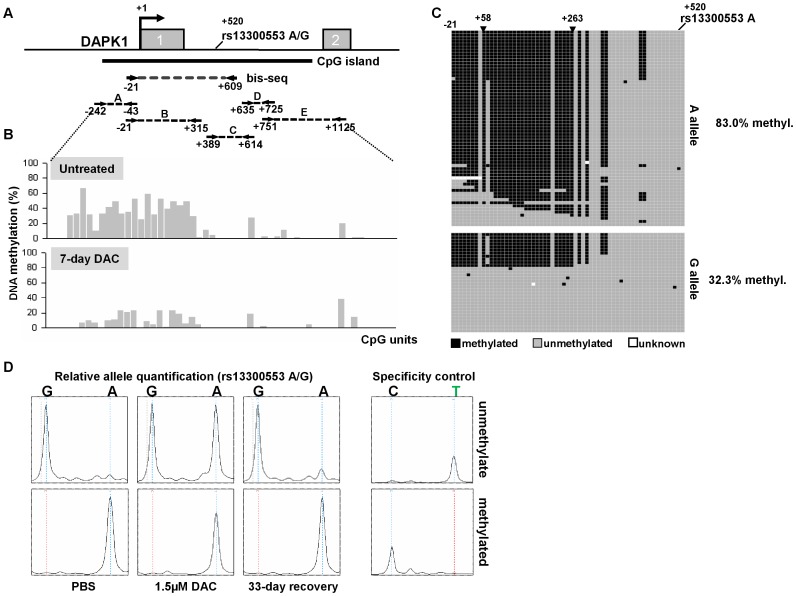
Allele-specific DNA methylation (ASM) contributes to ASE of *DAPK1* in Granta-519 cells. (A) Scheme of the *DAPK1* promoter region and the associated CpG island. Grey boxes display the first 2 exons of *DAPK1*. Nucleotide positions are given relative to *DAPK1* transcriptional start site (TSS). Dashed lines represent positions of the investigated regions/amplicons. (B) Quantitative DNA methylation analysis of the *DAPK1* gene 5′ region (amplicons A–E) in untreated and 5-aza-2′-deoxycytidine (DAC)-treated Granta-519 cells was performed using the MassARRAY-based MassCleave method. Bars represent quantitative DNA methylation values (%) at single CpG units. (C) Bisulfite-sequencing of the *DAPK1* 5′ region including the SNP rs13300553 (G/A) used for allelic separation in Granta-519 cells. Sequenced clones carrying A at the respective SNP site (+520) are grouped in the upper panel, the G alleles are displayed in the lower panel. Black boxes represent single-CpG methylation, grey boxes represent unmethylated CpGs, white boxes stand for missing data. Methylation levels are calculated over the area between +58 and +263 in both allele groups. (D) Detection of ASM by separate amplification of either the unmethylated or methylated alleles by methylation-specific PCR on bisulfite-converted genomic DNA. Genotype distribution between the differentially methylated alleles was performed by SNuPE/MALDI-TOF. Untreated Granta-519 (PBS), 7-day treatment with the DNMT inhibitor 5-aza-2′-deoxycytidine (DAC), and assessment of ASM after 33 days of withdrawal of DAC (33-day recovery) are shown. The right panel shows the assessment of a CpG dinucleotide as specificity control (see results section for detailed explanation).

To directly prove that allele-specific promoter methylation was associated with *DAPK1* ASE in Granta-519 cells, we quantitatively assessed DNA methylation in the *DAPK1* 5′ upstream region. While MEC-1 cells not expressing *DAPK1* exhibited almost complete DNA methylation from 200 bp upstream of TSS to exon 2 (**Figure S4**), a region of restricted methylation could be detected in ASE-positive Granta-519 at the end of exon 1 (**Figure 4A and 4B**). Quantitative assessment showed approximately 50% DNA methylation around exon 1, while most of the downstream region starting from intron 1 was unmethylated. Utilizing an informative SNP (rs13300553) in close vicinity to the 50% methylated region, we performed bisulfite sequencing to investigate allele-specific methylation patterns. The rs13300553 SNP A allele that represents the transcriptionally repressed allele showed 83.0% methylation whereas the G allele was methylated at considerably lower levels (∼32.3%) in the region of interest (**Figure 4C**). In EHEB cells exhibiting almost monoallelic expression, we found a similar separation in completely unmethylated and (almost) fully methylated alleles at the same region that exhibited ASM in Granta-519 cells (**Figure S7A and S7B**). However, as the SNP rs13300553 was not heterozygous in this cell line and other informative SNPs could not be detected between position −20 and +600, a clear allelic separation was not possible despite the strong evidence for two distinct allele populations. In JVM-2 cells with perfectly balanced *DAPK1* transcription, DNA methylation was entirely absent (**Figure S7C**). In order to quantitatively confirm the allele-specific promoter methylation (ASM), we designed a methylation-specific genotyping assay based on the SNuPE method (ASM-SNuPE). This method was used to determine the SNP ratio between the amplification of unmethylated and methylated alleles. Unmethylated and methylated amplicons were specifically amplified from bisulfite-treated DNA using PCR with primers specific for unmethylated or methylated template (UMSP/MSP) (**Figure 4D**). The primer design was based on differentially methylated CpGs as determined by the previous methylation results. We used an extension primer as an amplification specificity control to ensure for strict separation of methylated and unmethylated alleles. Quantitative genotyping of the SNP site rs13300553 in Granta-519 showed a strong enrichment of the G allele in the unmethylated fraction, whereas the A genotype almost exclusively appeared in the methylated alleles. DAC treatment increased the appearance of the A allele in the unmethylated fraction, indicating loss of methylation of this allele. Withdrawal of DAC restored the allele-specific methylation after cultivation for one month. Taken together, these experiments show that in Granta-519 *DAPK1* ASE and ASM are functionally related.

## Discussion

*DAPK1* is proposed to be a tumor suppressor gene in CLL. In a recent study, a rare sequence variant associated with early disease onset in a large CLL family was shown to reduce *DAPK1* expression on one allele to 25% of the normal level, resulting in ASE [8]. Other genetic alterations were not identified to affect *DAPK1* in CLL. In the present study, we investigated the extent of germline ASE of the *DAPK1* gene in CLL under the hypothesis that this might be a possible novel mechanism predisposing individuals to CLL.

The association of ASE with tumor predisposition was first reported in 2001 [11]. However, prevalence and mechanisms of ASE in tumorigenesis remain largely unknown. Recently it was demonstrated that reduced levels of the tumor suppressor gene *APC* are associated with pronounced predisposition to familial adenomatous polyposis [31]. Linkage analysis in these families showed that the allele with reduced APC expression was linked to the disease; however, a genetic alteration that might explain the reduced expression of *APC* could not be identified. This work was followed by a study by Valle et al. [13], demonstrating ASE of *TGFBR1* in 10–20% of colorectal cancer patients as opposed to 1–3% in control populations. Reduced *TGFBR1* expression affects the SMAD–mediated TGF-beta signaling. The authors report that ASE is inherited in familial cases and occurs also in sporadic cases of colorectal cancer. Two major haplotypes associated with the reduced expression of *TGFBR1* were reported, however no mutation that may explain this phenomenon was detected. In subsequent studies this group revised the reported frequencies of ASE in *TGFBR1* to fewer cases and the authors conclude that improved quantitative techniques are required for reliable ASE detection [32], [33]. We established a quantitative SNuPE/MALDI-TOF-based approach for ASE assessment that is sensitive and robust. Furthermore, the high-throughput capability of this assay enables investigation of larger cohorts e.g. of large epidemiological studies. Using this novel approach, we observed *DAPK1* ASE in non-malignant (germline) cells in 14% of CLL cases but not in a control population evoking a novel potential mechanism for predisposition to CLL. A trend toward germline ASE positive patients being of younger age at disease onset/age at diagnosis could substantiate a predisposing role for *DAPK1* ASE. We did not detect any correlation of *DAPK1* ASE with familial occurrence of CLL, which has a reported prevalence of 5–10%, although the power to detect such a correlation in this cohort is low. Systematic assessment of such information in a prospective manner would be needed to draw valid conclusions. The need of heterozygosity at specific exonic SNPs and the rather low frequencies of ASE cases led to the identification of a rather small number of ASE positive patients (17 out of a collective of 303 patients who were initially included in the study). Furthermore, a prospective investigation of *DAPK1* ASE in healthy individuals with monoclonal B cell lymphocytosis (MBL), a potential precursor of CLL that shows a prevalence of up to 3.5% in the entire population, would allow for a more accurate assessment of the predisposing character of *DAPK1* ASE [34].

So far the mechanisms that cause allelic imbalance of mRNA expression are not clear. In our previous work, a disease haplotype and mutation could be identified which segregated with the CLL phenotype in a large family [8]. However, in the general population this mutation is extremely rare. Reports about ASE of both *BRCA1* and *BRCA2* to be associated with increased breast cancer risk [14] indicated that in some of the cases, ASE could be explained by mutations activating the nonsense mediated mRNA decay. In the majority of cases, however, ASE remained mechanistically unexplained. In a report implicating the association of *CDH1* ASE with hereditary diffuse gastric cancer [35], one ASE-positive proband showed an unusual pattern of allele-specific methylation in the promoter. To elucidate the potential mechanisms of *DAPK1* ASE in CLL, we investigated a CLL-related leukemic cell line model. Prompted by the observation of extensive epigenetic silencing by DNA methylation of *DAPK1* in the clonal malignant B cells of CLL patients, we hypothesized a role for an underlying epigenetic cause of ASE in the non-malignant (germline) cells. In contrast to the previously reported unidirectional expression imbalances of *TGFBR1*, *DAPK1* ASE was found to be bi-directional implicating shifts to either allele. This could support the role of DNA methylation as underlying silencing event potentially induced from a different locus in *trans*. In Granta-519 cells, which showed a pronounced allelic mRNA expression imbalance without any copy number variations in the region of the *DAPK1* gene, promoter DNA methylation levels of approximately 50% were observed. An allele-specific distribution of DNA methylation was associated with the repressed allele. Furthermore, we could show that after erasure of DNA methylation at this locus by a DNA hypomethylating agent, re-establishment of ASE occurred exclusively at the initially repressed allele. This indicates that epigenetic mechanisms could cause ASE of *DAPK1* in CLL-relevant cell line models. We postulated an underlying genetic mutation as a cause for the allelic restriction of DNA methylation in ASE-positive Granta-519. However, sequencing up to approximately 6 kb upstream of *DAPK1* TSS did not reveal any genetic variation. Similarly, we analyzed germline material from ASE-positive patients for allele-specific epigenetic marks and used patients with perfect allelic balance as control. We could not detect any genetic aberrations in the *DAPK1* 5′ upstream regulatory region. Interestingly, we observed significantly elevated DNA methylation in ASE-positive cases around the transcriptional start site, which is in concordance with ASM observed in Granta-519 cells and might point towards an epigenetic cause for ASE. The primary genetic basis might act in *trans* far from the target and may be difficult to detect. However, the methylation differences were subtle and it remains mechanistically unclear how these differences are established and whether they might be causative for ASE. Epigenetic mechanisms have the potential of modulating gene expression, but so far they have not been thoroughly investigated as a potential mechanism for ASE. Exceptions are epimutations identified in *MLH1* or *MSH2* leading to gene silencing and predisposition in hereditary forms of colorectal cancer [23]. For some of these epimutations, genetic alterations have been described that can trigger epigenetic events. For example, it has been shown that heterozygous germline deletions of the last exon of *TACSTD1*, a gene directly upstream of *MSH2*, resulted in extension of the transcription into the promoter of *MSH2*, thereby triggering by an unknown mechanism subsequent epigenetic alteration of the *MSH2* promoter [36]. Currently we do not know if and how germline epigenetic alterations could affect *DAPK1* expression and thereby might contribute to the predisposing mechanism. Additional molecular mechanisms causing ASE could be nonsense-mediated mRNA decay, due to a mutation in the target gene, which seems to be a rare event. Another possible mechanism could be the modulation of miRNA binding due to sequence variations or sequence alterations affecting the promoter activity of cancer genes. High throughput genome analysis has uncovered copy number variations occurring throughout genomes of healthy individuals. These variations could result in allelic imbalances of gene expression. Similarly, differentially methylated regions other than at imprinted regions can have similar effects on gene expression. The extent to which these mechanisms participate in ASE and cancer predisposition needs to be determined in future studies. Furthermore, prospective trials are needed to confirm these findings and to extend a predisposing role of *DAPK1* ASE to non-malignant CLL precursor states like monoclonal B-cell lymphocytosis.

## Supporting Information

Figure S1 **Accurate quantification of** ***DAPK1*** **ASE investigating all four common exonic SNPs.** Standard curves for plasmid based standards displaying allelic ratios from 1∶50 to 50∶1 and correlation with idealized ratios. (A) SNP rs3118863, DAPK1 exon 26, (B) SNP rs3818584, DAPK1 exon 16, (C) SNP rs36207428, DAPK1 exon 3. (D) SNP rs1056719, DAPK1 exon 26.
(TIF)

Figure S2 **Detection sensitivity for quantitative genotyping of rs1056719.** (A) Standard curves for plasmid molecular standard (A: 30,000 template plasmid copies, B: 300 template plasmid copies) and comparison with ideal linear correlation. Standard deviations are given for 4 replicate measurements.
(TIF)

Figure S3 ***DAPK1*** **ASE in CD19 depleted PBMC samples from CLL patients.** (A) 120 CLL cases, 11 CD19 depleted (contaminating CD19+ population less than 2%) and 63 controls were analyzed for *DAPK1* ASE using the informative SNP rs1056719 (G/A) as outlined previously. Allelic ratios (in relation to the G allele) of *DAPK1* mRNA were measured with the outlined SNuPE/MALDI-TOF-based method.
(TIF)

Figure S4 **MEC-1 cells are fully methylated at the CpG island of the** ***DAPK1*** **5′ region.** (A) Scheme of the *DAPK1* promoter region and the associated CpG island. Grey boxes display the first 2 exons of *DAPK1*. Nucleotide positions are given relative to the *DAPK1* transcriptional start site. Dashed lines represent positions of investigated regions/amplicons. (B) Quantitative DNA methylation analysis of the *DAPK1* gene 5′ region (amplicons A–E) in untreated, control (PBS)-treated and 5-aza-2′-deoxycytidine (DAC)-treated MEC-1 cells was performed using the MassCleave method. Bars represent quantitative DNA methylation values (%) at single CpG units.
(TIF)

Figure S5 **Detection of** ***DAPK1*** **ASE by conventional Sanger sequencing.** (A) Sequences from 12 single clones of ligated PCR products of the *DAPK1* cDNA. rs1056719 indicates the polymorphic site, the arrows represent the cloning primers and indicate the sequencing direction. (B) Chromatograms representing the genomic region around the polymorphic site rs1056719 in Granta-519 cells. The upper panel displays the cDNA, the lower panel displays the genomic DNA as balancing control. (C) Chromatograms representing the polymorphic site rs3818584 in EHEB cells according to figure 3B.
(TIF)

Figure S6 **Re-balancing of** ***DAPK1*** **mRNA expression in Granta-519 cells upon inhibition of DNA methyltransferases assessed by Sanger sequencing.** (A) Chromatograms representing the genomic region around the polymorphic site rs1056719 in Granta-519 cells after seven days of control treatment with the solvent PBS. (B) Rebalancing after seven days of pulsed treatment with 1.5 µM DNA methyltransferase inhibitor 5-aza-2′-deoxycytidine (DAC). (C) Re-constitution of the allelic imbalance after 1.5 µM DAC treatment for seven days and consecutive withdrawal of the compound for 33 days.
(TIF)

Figure S7 **Allelic DNA methylation in lymphoid cell lines with allele-specific expression of the** ***DAPK1*** **gene.** (A) Scheme of the *DAPK1* promoter region and the associated CpG island. Grey boxes display the first two exons of *DAPK1*. Nucleotide positions are given relative to the *DAPK1* transcriptional start site. The dashed line represents the amplicon analyzed by bisulfite sequencing. This region exhibited extensive *DAPK1* allele-specific DNA methylation in Granta-519 cells. (B, C) Bisulfite-sequencing of the *DAPK1* 5′ region in JVM-2 (no ASE) and EHEB (monoallelic expression) cells. As a heterozygous SNP could be detected in neither cell line between −20 and +600 bp, a clear allelic separation is not possible. Red boxes represent single-CpG methylation, blue boxes represent unmethylated CpGs, white boxes stand for missing data. Methylation levels are calculated in percent for each CpG dinucleotide.
(TIF)

Table S1 **Oligonucleotides and primers.**
(DOC)
