# Supplementary figures and images for: Germline Allele-Specific Expression of DAPK1 in Chronic Lymphocytic Leukemia
Source: PLoS One. 2013 Jan 28;8(1):e55261. doi: 10.1371/journal.pone.0055261 (PMC3557246; doi:10.1371/journal.pone.0055261)

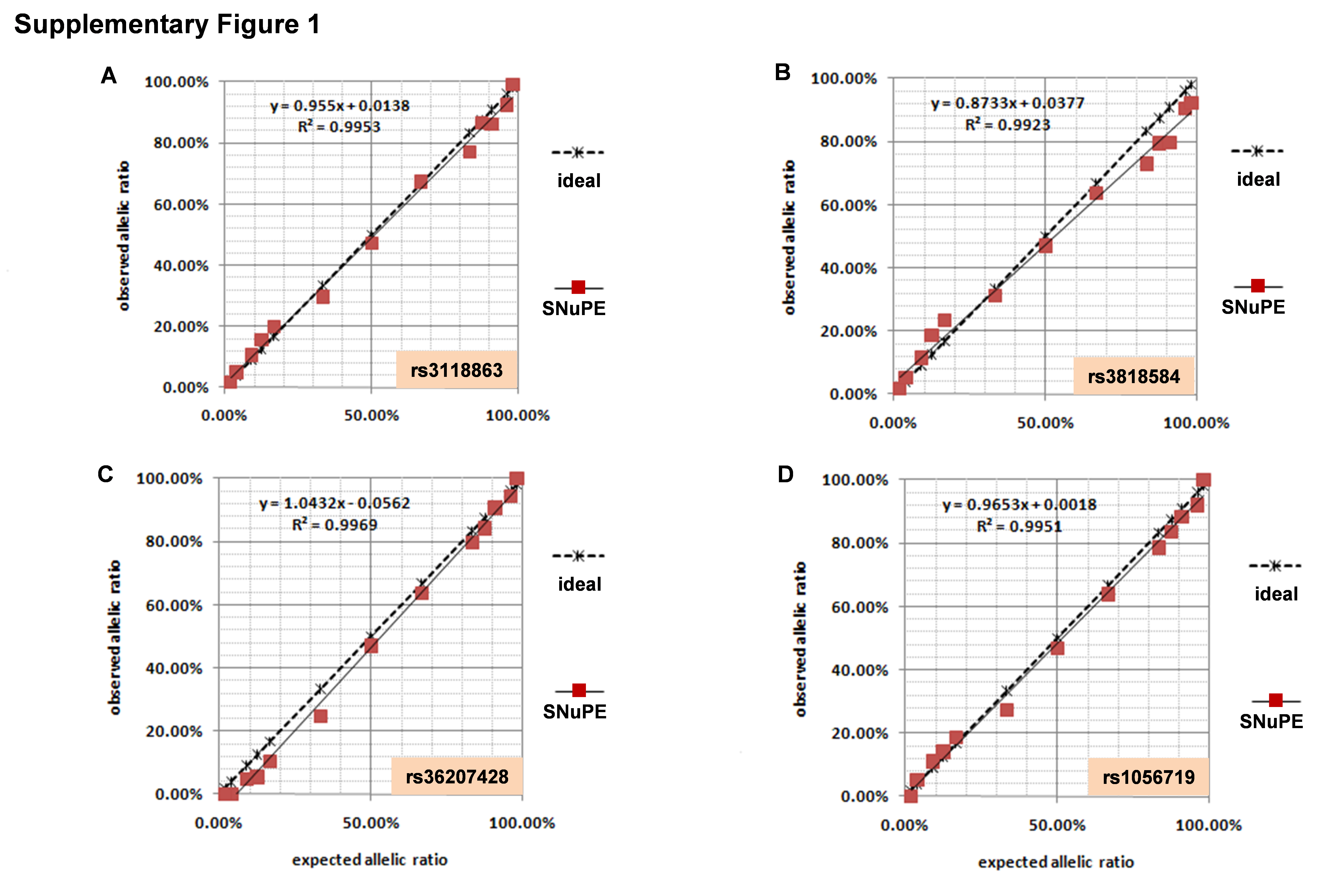

Supplement: Figure S1 — Accurate quantification of DAPK1 ASE investigating all four common exonic SNPs. Standard curves for plasmid based standards displaying allelic ratios from 1∶50 to 50∶1 and correlation with idealized ratios. (A) SNP rs3118863, DAPK1 exon 26, (B) SNP rs3818584, DAPK1 exon 16, (C) SNP rs36207428, DAPK1 exon 3. (D) SNP rs1056719, DAPK1 exon 26. (TIF) [file pone.0055261.s001.tif]

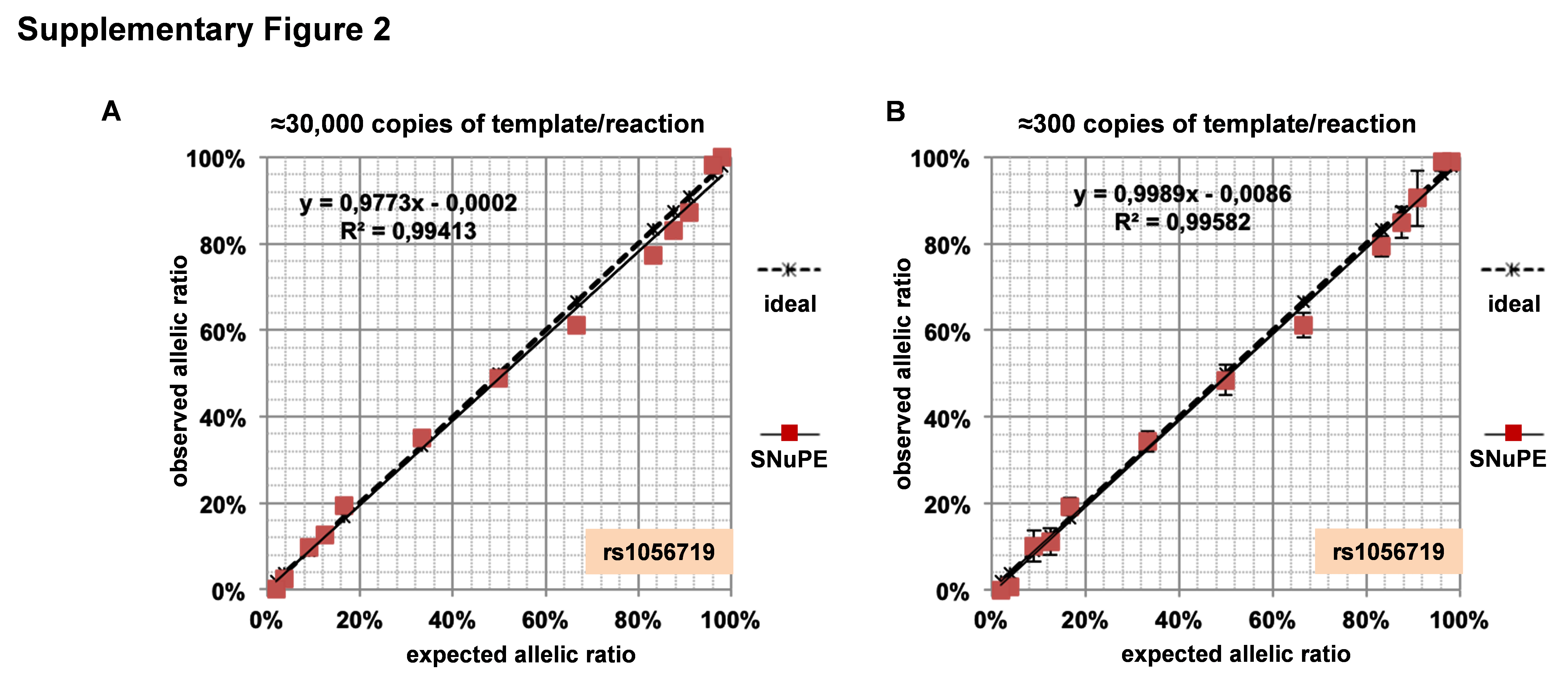

Supplement: Figure S2 — Detection sensitivity for quantitative genotyping of rs1056719. (A) Standard curves for plasmid molecular standard (A: 30,000 template plasmid copies, B: 300 template plasmid copies) and comparison with ideal linear correlation. Standard deviations are given for 4 replicate measurements. (TIF) [file pone.0055261.s002.tif]

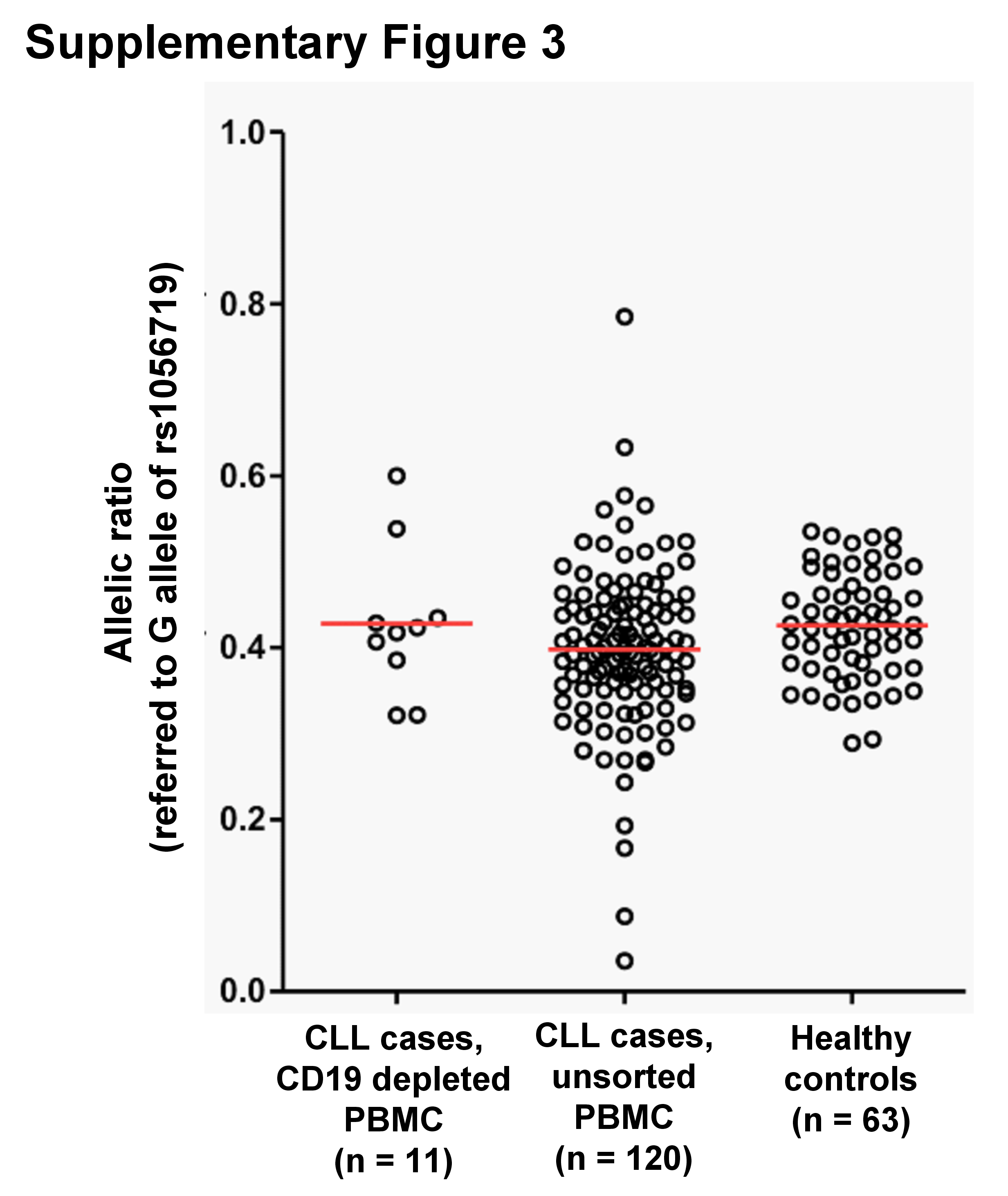

Supplement: Figure S3 — DAPK1 ASE in CD19 depleted PBMC samples from CLL patients. (A) 120 CLL cases, 11 CD19 depleted (contaminating CD19+ population less than 2%) and 63 controls were analyzed for DAPK1 ASE using the informative SNP rs1056719 (G/A) as outlined previously. Allelic ratios (in relation to the G allele) of DAPK1 mRNA were measured with the outlined SNuPE/MALDI-TOF-based method. (TIF) [file pone.0055261.s003.tif]

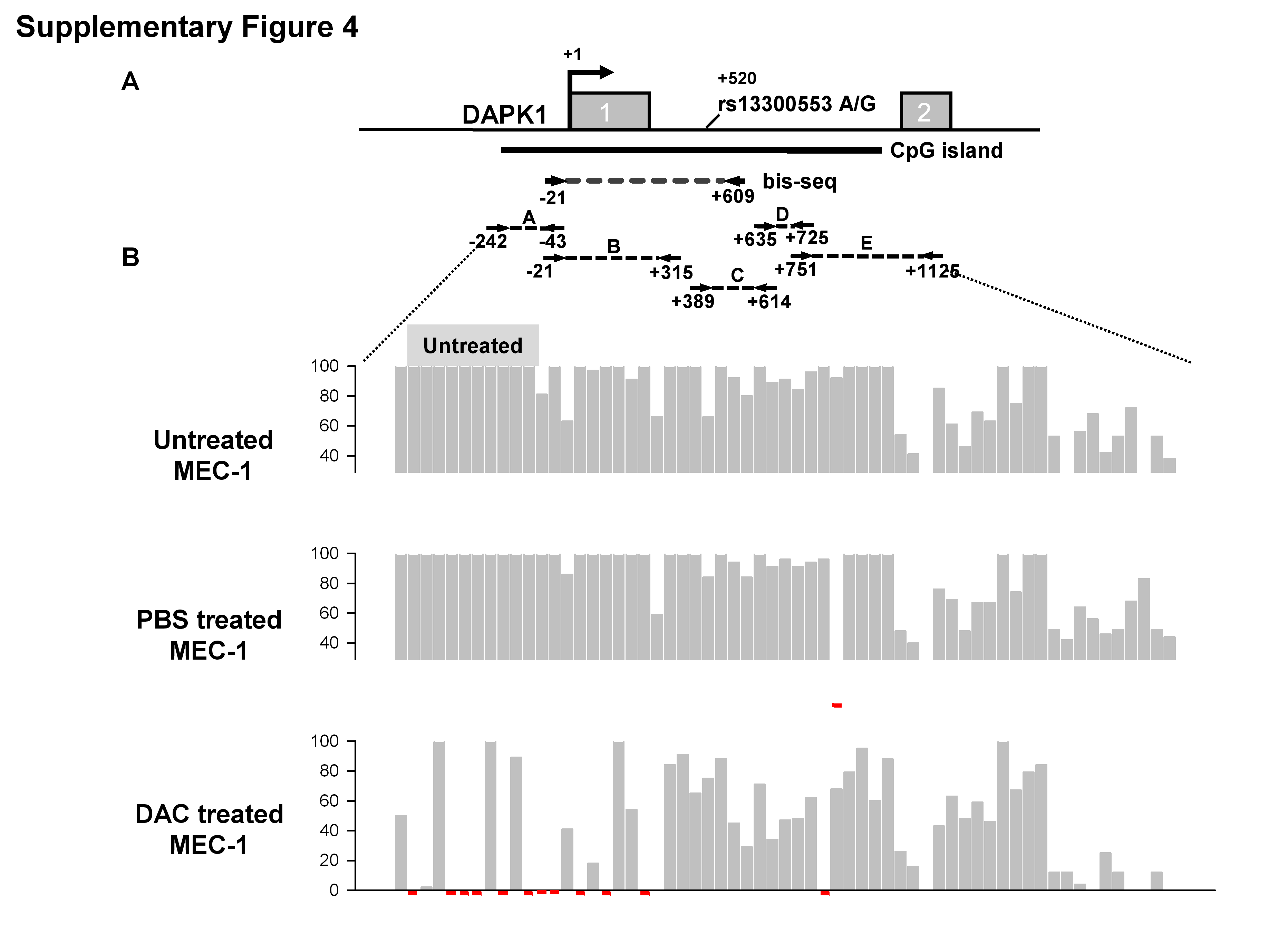

Supplement: Figure S4 — MEC-1 cells are fully methylated at the CpG island of the DAPK1 5′ region. (A) Scheme of the DAPK1 promoter region and the associated CpG island. Grey boxes display the first 2 exons of DAPK1. Nucleotide positions are given relative to the DAPK1 transcriptional start site. Dashed lines represent positions of investigated regions/amplicons. (B) Quantitative DNA methylation analysis of the DAPK1 gene 5′ region (amplicons A–E) in untreated, control (PBS)-treated and 5-aza-2′-deoxycytidine (DAC)-treated MEC-1 cells was performed using the MassCleave method. Bars represent quantitative DNA methylation values (%) at single CpG units. (TIF) [file pone.0055261.s004.tif]

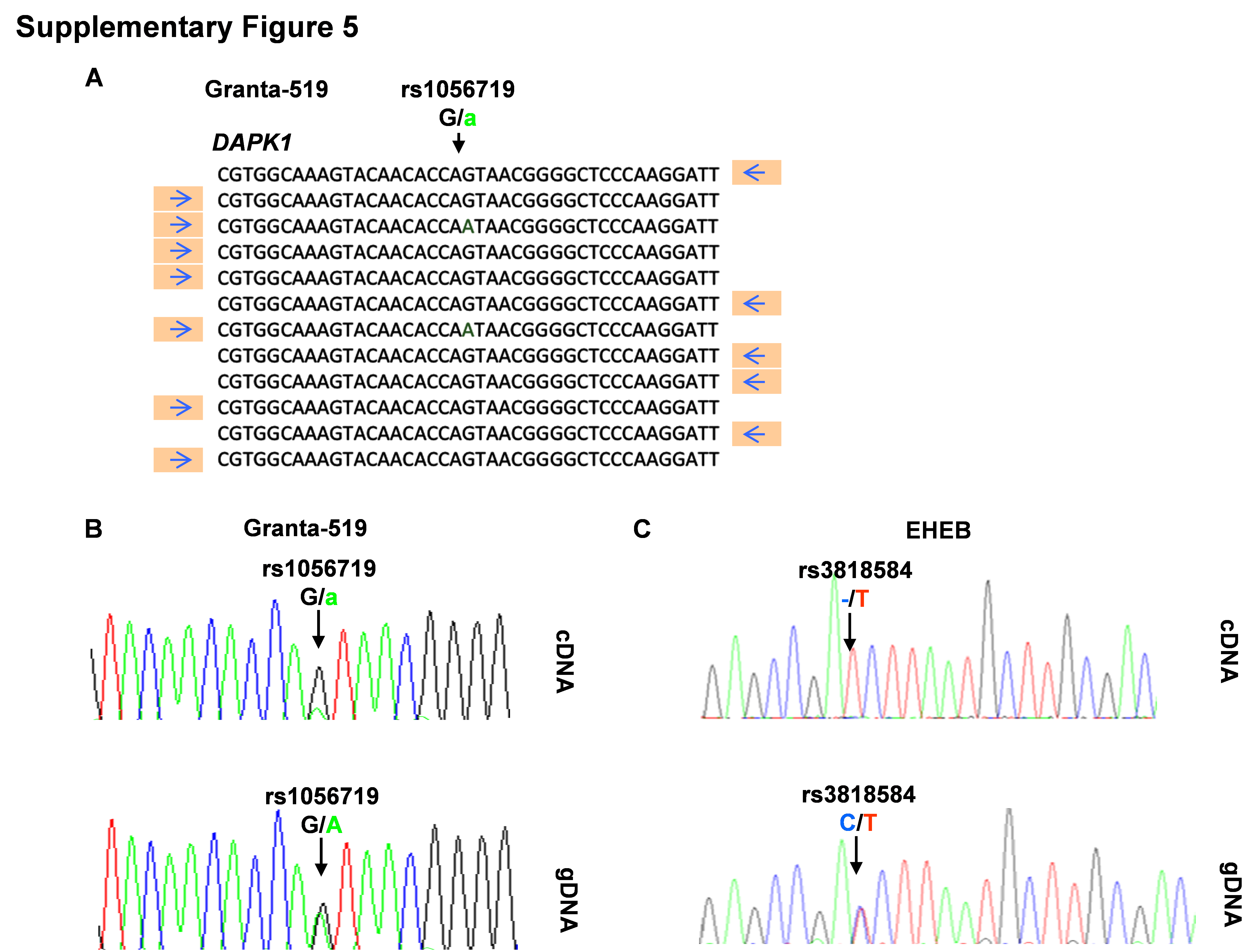

Supplement: Figure S5 — Detection of DAPK1 ASE by conventional Sanger sequencing. (A) Sequences from 12 single clones of ligated PCR products of the DAPK1 cDNA. rs1056719 indicates the polymorphic site, the arrows represent the cloning primers and indicate the sequencing direction. (B) Chromatograms representing the genomic region around the polymorphic site rs1056719 in Granta-519 cells. The upper panel displays the cDNA, the lower panel displays the genomic DNA as balancing control. (C) Chromatograms representing the polymorphic site rs3818584 in EHEB cells according to figure 3B. (TIF) [file pone.0055261.s005.tif]

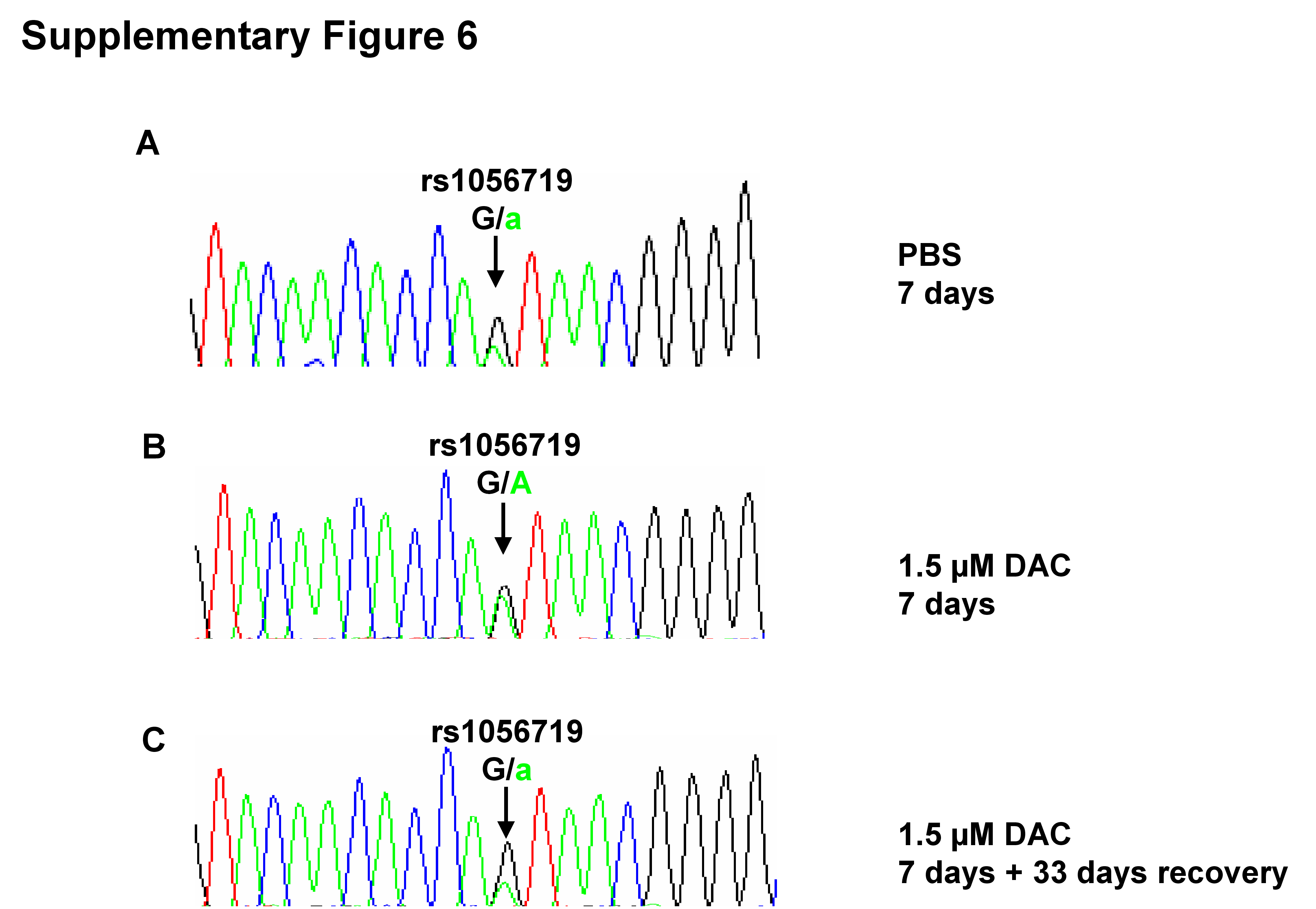

Supplement: Figure S6 — Re-balancing of DAPK1 mRNA expression in Granta-519 cells upon inhibition of DNA methyltransferases assessed by Sanger sequencing. (A) Chromatograms representing the genomic region around the polymorphic site rs1056719 in Granta-519 cells after seven days of control treatment with the solvent PBS. (B) Rebalancing after seven days of pulsed treatment with 1.5 µM DNA methyltransferase inhibitor 5-aza-2′-deoxycytidine (DAC). (C) Re-constitution of the allelic imbalance after 1.5 µM DAC treatment for seven days and consecutive withdrawal of the compound for 33 days. (TIF) [file pone.0055261.s006.tif]

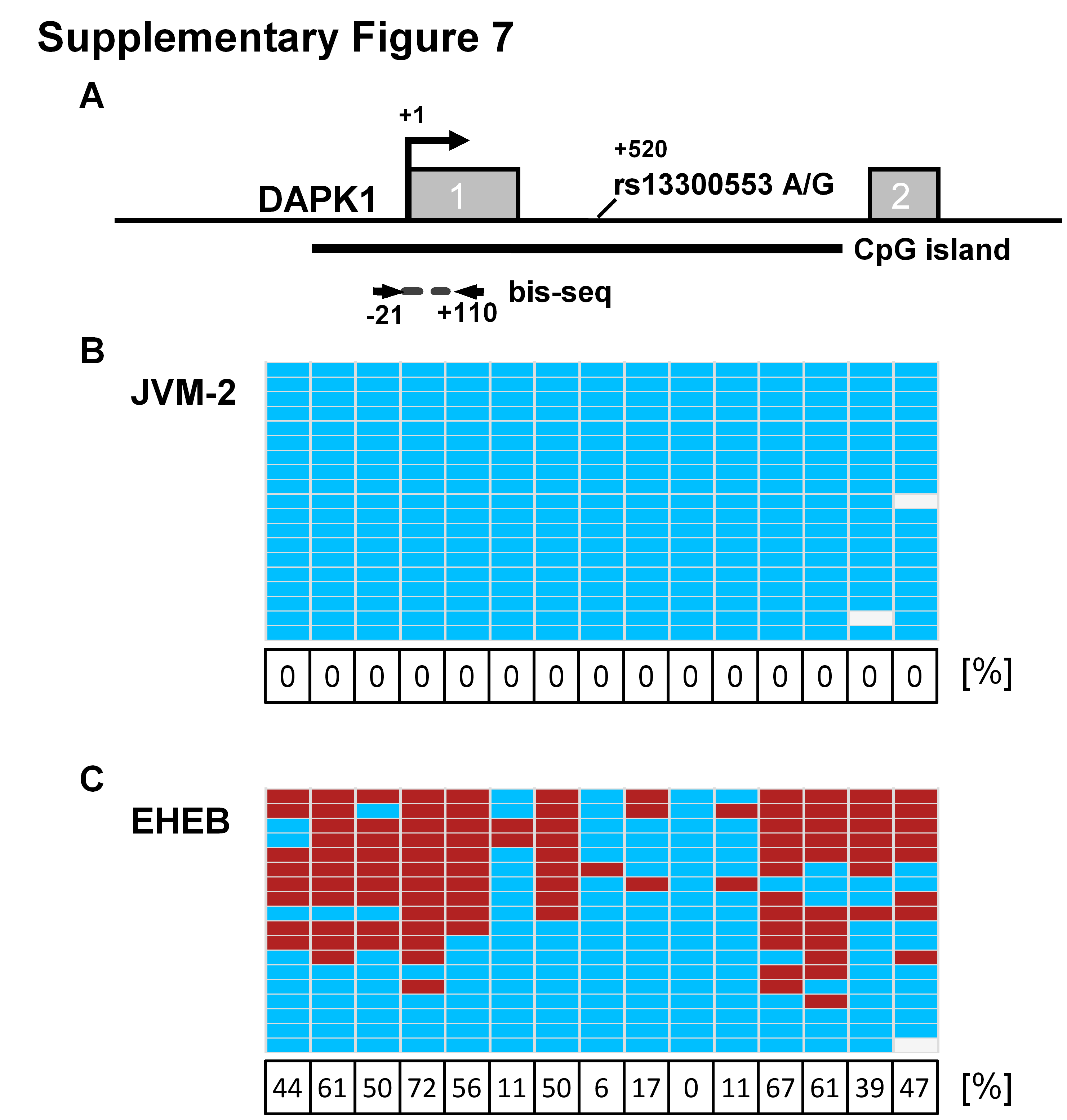

Supplement: Figure S7 — Allelic DNA methylation in lymphoid cell lines with allele-specific expression of the DAPK1 gene. (A) Scheme of the DAPK1 promoter region and the associated CpG island. Grey boxes display the first two exons of DAPK1. Nucleotide positions are given relative to the DAPK1 transcriptional start site. The dashed line represents the amplicon analyzed by bisulfite sequencing. This region exhibited extensive DAPK1 allele-specific DNA methylation in Granta-519 cells. (B, C) Bisulfite-sequencing of the DAPK1 5′ region in JVM-2 (no ASE) and EHEB (monoallelic expression) cells. As a heterozygous SNP could be detected in neither cell line between −20 and +600 bp, a clear allelic separation is not possible. Red boxes represent single-CpG methylation, blue boxes represent unmethylated CpGs, white boxes stand for missing data. Methylation levels are calculated in percent for each CpG dinucleotide. (TIF) [file pone.0055261.s007.tif]
