# Supplementary material for: Germline Allele-Specific Expression of DAPK1 in Chronic Lymphocytic Leukemia
Source: PLoS One. 2013 Jan 28;8(1):e55261. doi: 10.1371/journal.pone.0055261 (PMC3557246; doi:10.1371/journal.pone.0055261)
Supplement: Table S1 — Oligonucleotides and primers. (DOC) [file pone.0055261.s008.doc]

**Supplementary Table 1**

| **Primer name/application** | **Sequence ( 5'→ 3')** | **Gene/SNP** | **Template** |
| --- | --- | --- | --- |
| **Sequencing** | | | |
| DEx3_F | GCAGGAAAACGTGGATGATT | DAPK1/rs36207428 | cDNA |
| DEx3_R | CTGGATCTCCTTCAGGATGC | DAPK1/rs36207428 | cDNA |
| gDEx3_F | TGGACAGTTTGCGGTTGTGAAG | DAPK1/rs36207428 | gDNA |
| gDEx3_R | AGGATGACGTCCGTCTTGTTCTC | DAPK1/rs36207428 | gDNA |
| DEx16_F | CTTCGGCTCAAATCCCAATA | DAPK1/rs3818584 | cDNA |
| DEx16_R | GCCGTGCCTGTCTTGATAAT | DAPK1/rs3818584 | cDNA |
| gDEx16_F | ACGGCTATTACTCTGTGGCCAAAG | DAPK1/rs3818584 | gDNA |
| gDEx16_R | AACAAACTGAGGAGTCTTGTGGTC | DAPK1/rs3818584 | gDNA |
| DEx26_Fa | GACCTGAACCTCCTCACTCG | DAPK1/rs1056719 | cDNA&gDNA |
| DEx26_R | GCCTTCAGCAAAAAGTCTGC | DAPK1/rs1056719 | cDNA&gDNA |
| DEx26_F | ATCCACCAGCAAAGCACA | DAPK1/rs3118863 | cDNA&gDNA |
| DEx26_Rb | CCATGACGTTCTCAATGGTG | DAPK1/rs3118863 | cDNA&gDNA |
| **Genotyping** | | | |
| rs13300553.1 | ACGTTGGATGAGCAAGGGAGGGAAGGGAG | DAPK1/rs13300553 | gDNA |
| rs13300553.2 | ACGTTGGATGACCAGGCGCTTTGTGTCGG | DAPK1/rs13300553 | gDNA |
| rs13300553.UEP | CACCGAGGCGCAGCAGT | DAPK1/rs13300553 | PCR products |
| rs1329600.1 | ACGTTGGATGTGACACATTGCTAAGCTGCC | DAPK1/rs1329600 | gDNA |
| rs1329600.2 | ACGTTGGATGGGGATCCCAGTACACATCTC | DAPK1/rs1329600 | gDNA |
| rs1329600.UEP | TCTAGGGAAACACGGTTCTT | DAPK1/rs1329600 | PCR products |
| rs1035261.1 | ACGTTGGATGGAGAGGTGAGAAATACAGTC | DAPK1/rs1035261 | gDNA |
| rs1035261.2 | ACGTTGGATGTTCTCCCAACAGATGTATTC | DAPK1/rs1035261 | gDNA |
| rs1035261.UEP | AAATAACAACAACAACAAAAAAA | DAPK1/rs1035261 | PCR products |
| rs1035260.1 | ACGTTGGATGCAAAAGGGTCTTGAGAGGTG | DAPK1/rs1035260 | gDNA |
| rs1035260.2 | ACGTTGGATGGCATTTTCCCAGATGAATCAC | DAPK1/rs1035260 | gDNA |
| rs1035260.UEP | TCAAAAATAACAACAACAACAA | DAPK1/rs1035260 | PCR products |
| **Allele-specific expression** | | | |
| rs1056719-1 | ACGTTGGATGCCTTCTCGCCATGAACTTAG | DAPK1/rs1056719 | cDNA&gDNA |
| rs1056719_2 DNA | ACGTTGGATGCTCCCTCAGTTTGGACATGA | DAPK1/rs1056719 | cDNA&gDNA |
| rs36207428-1 | ACGTTGGATGGGTTGTGAAGAAATGCCGTG | DAPK1/rs36207428 | cDNA&gDNA |
| rs36207428_2 DNA | ACGTTGGATGATAGACCTCGTGCAGGGTGA | DAPK1/rs36207428 | cDNA&gDNA |
| rs3118863-1 | ACGTTGGATGAGGCAGCACTTGATCTTCTC | DAPK1/rs3118863 | cDNA&gDNA |
| rs3118863_1 DNA | ACGTTGGATGATCCACCAGCAAAGCACAG | DAPK1/rs3118863 | cDNA&gDNA |
| rs3818584-1 | ACGTTGGATGACGACATCGTGGAGTGTCTG | DAPK1/rs3818584 | cDNA&gDNA |
| rs3818584_2 DNA | ACGTTGGATGTTGTTATCTTAGGGAAACAAACTGA | DAPK1/rs3818584 | gDNA |
| rs3818584_2 cDNA | ACGTTGGATGAGTATTGCCGTGCCTGTCTT | DAPK1/rs3818584 | cDNA |
| rs1056719-UEP | CCTTGGGAGCCCCGTTA | DAPK1/rs1056719 | PCR products |
| rs36207428-UEP | GATGAATTTGGCGGCATA | DAPK1/rs36207428 | PCR products |
| rs3118863-UEP | GGCATTGAGGTCCAGGTCCG | DAPK1/rs3118863 | PCR products |
| rs3818584-UEP | CGTCATTGTCGCAAGCATTAAG | DAPK1/rs3818584 | PCR products |
| **Quantitative DNA methylation analysis** | | | |
| DAPK1-0.2_10F | AGGAAGAGAGAGTTTAGTAATGTGTTATAGGTG | DAPK1 | BT-DNA |
| DAPK1-0.2_T7R | CAGTAATACGACTCACTATAGGGAGAAGGCTACCAATAAAAACCCTACAAAC | DAPK1 | BT-DNA |
| DAPK1-1_10F | AGGAAGAGAGGTTTGTAGGGTTTTTATTGGT | DAPK1 | BT-DNA |
| DAPK1-1_T7R | CAGTAATACGACTCACTATAGGGAGAAGGCTCCCTAACTAAAAAAACAAAAACT | DAPK1 | BT-DNA |
| DAPK1-3_10F | AGGAAGAGAGGAGTAATTGGGAAGGTTAAGG | DAPK1 | BT-DNA |
| DAPK1-3_T7R | CAGTAATACGACTCACTATAGGGAGAAGGCTAAACAATCCCCAAAACCACATTC | DAPK1 | BT-DNA |
| DAPK1-5_10F | AGGAAGAGAGTAGGAATGTGGTTTTGGGGATTG | DAPK1 | BT-DNA |
| DAPK1-5_T7R | CAGTAATACGACTCACTATAGGGAGAAGGCTCCTCACTAAAAACAATCTCTCTCCA | DAPK1 | BT-DNA |
| DAPK1-6_10F | AGGAAGAGAGTGGAGAGAGATTGTTTTTAGTGAGG | DAPK1 | BT-DNA |
| DAPK1-6_T7R | CAGTAATACGACTCACTATAGGGAGAAGGCTTCATACATCAATCTCCAATCCTTTTA | DAPK1 | BT-DNA |
| **Bisulfite-sequencing** | | | |
| DAPK1-BS_SNP_F1a | GTTTGTAGGGTTTTTATTGGT | DAPK1/rs13300553 | BT-DNA |
| DAPK1-BS_SNP_R | TCCCCAAAACCACATTCCTAAC | DAPK1/rs13300553 | BT-DNA |
| **Allele-specific DNA methylation** | | | |
| PQR_F | TAGTGGTAGGGTTTGGGGTTGGT | DAPK1/rs13300553 | BT-DNA |
| DAPK1 MSP_F1 | TTTTCGATAGCGTTTCGGAGGGATC | DAPK1/rs13300553 | BT-DNA |
| SNP1_R | AACAATCCCCAAAACCACATTCCTAAC | DAPK1/rs13300553 | BT-DNA |
| rs13300553 dI-UEP | CCACCIAAACICAACAAT | DAPK1/rs13300553 | UMSP&MSP products |
| DAPK1_ASM_ctrl_1 | GGGTGAGTAGTTAGG | DAPK1/rs13300553 | UMSP&MSP products |
| **mRNA expression analysis** | | | |
| DAPKR2P86_F | GCAGGAAAACGTGGATGATT | DAPK1 | cDNA |
| DAPKR2P86_R | CATTTCTTCACAACCGCAAA | DAPK1 | cDNA |
| NS-P8R2_F | GCCAGATTGTTTCGGTCTGT | DAPK1 | cDNA |
| NS-P8R2_R | GGTTGGGTCCATTGAGCTT | DAPK1 | cDNA |
| RT-ACTB-F | ATTGGCAATGAGCGGTTC | β-ACTIN | cDNA |
| RT-ACTB-R | GGATGCCACAGGACTCCAT | β-ACTIN | cDNA |
| rt-GAPDH-R | GCCCAATACGACCAAATCC | GAPDH | cDNA |
| rt-GAPDH-F | AGCCACATCGCTCAGACAC | GAPDH | cDNA |
| rt-HPRT1-F | TGACCTTGATTTATTTTGCATACC | HPRT | cDNA |
| rt-HPRT1-R | CGAGCAAGACGTTCAGTCCT | HPRT | cDNA |
| h-CEBPb_F | GACAAGCACAGCGACGAGTA | C/EBP-β | cDNA |
| h-CEBPb_R | AGCTGCTCCACCTTCTTCTG | C/EBP-β | cDNA |

Abbreviations: F, forward; R, reverse; gDNA, genomic DNA; BT-DNA, bisulfite-treated genomic DNA.
